# Metformin Hydrochloride Significantly Inhibits Rotavirus Infection in Caco2 Cell Line, Intestinal Organoids, and Mice

**DOI:** 10.3390/ph16091279

**Published:** 2023-09-11

**Authors:** Rui Zhang, Cui Feng, Dandan Luo, Ruibo Zhao, Perumal Ramesh Kannan, Yuebang Yin, Muhammad Zubair Iqbal, Yeting Hu, Xiangdong Kong

**Affiliations:** 1 Institute for Smart Biomedical Materials, School of Materials Science & Engineering, Zhejiang Sci-Tech University, Hangzhou 310018, China; zhangrui0831@126.com (R.Z.); fengc93103@126.com (C.F.); 201810301008@mails.zstu.edu.cn (D.L.); rzhao@zstu.edu.cn (R.Z.); rameshclri07@gmail.com (P.R.K.); tonyerasmusyin@163.com (Y.Y.); zubair@zstu.edu.cn (M.Z.I.); 2 Zhejiang-Mauritius Joint Research Center for Biomaterials and Tissue Engineering, Zhejiang Sci-Tech University, Hangzhou 310018, China; 3 Department of Colorectal Surgery and Oncology, Key Laboratory of Cancer Prevention and Intervention, Ministry of Education, The Second Affiliated Hospital, Zhejiang University School of Medicine, Hangzhou 310030, China

**Keywords:** rotavirus, metformin hydrochloride, Caco2, intestinal organoid, sucking mice

## Abstract

Rotavirus is one of the main pathogens that causes severe diarrhea in children under the age of 5, primarily infecting the enterocytes of the small intestine. Currently, there are no specific drugs available for oral rehydration and antiviral therapy targeting rotavirus. However, metformin hydrochloride, a drug known for its antiviral properties, shows promise as it accumulates in the small intestine and modulates the intestinal microbiota. Therefore, we formulated a hypothesis that metformin hydrochloride could inhibit rotavirus replication in the intestine. To validate the anti-rotavirus effect of metformin hydrochloride, we conducted infection experiments using different models, ranging from in vitro cells and organoids to small intestines in vivo. The findings indicate that a concentration of 0.5 mM metformin hydrochloride significantly inhibits the expression of rotavirus mRNA and protein in Caco-2 cells, small intestinal organoids, and suckling mice models. Rotavirus infections lead to noticeable pathological changes, but treatment with metformin has been observed to mitigate the lesions caused by rotavirus infection in the treated group. Our study establishes that metformin hydrochloride can inhibit rotavirus replication, while also affirming the reliability of organoids as a virus model for in vitro research.

## 1. Introduction

Despite the availability of an effective vaccine, rotavirus remains a significant cause of mortality in children worldwide, resulting in approximately 215,000 deaths each year [1]. This highly contagious virus poses a substantial risk to infants and young children and exhibits strong resistance to environmental conditions, allowing it to cause systemic infections and invade multiple organs [2]. Notably, rotavirus infections have emerged as a leading cause of death, particularly among children who have undergone organ transplants, as these individuals had more severe symptoms of diarrhea after rotavirus infection [3]. Although possible treatments have been proposed, including immunotherapy, probiotic supplementation, traditional Chinese medicine, and natural compounds, the main method for treating rotavirus-induced diarrhea in clinical practice is still to replenish lost fluids and electrolytes, and there are currently no specific drugs targeting this virus [4]. Vaccines reduce rotavirus infection to a certain extent, but for countries with poor development, vaccination against rotavirus is subject to an enormous economic burden [5]. Therefore, rotavirus continues to pose a significant threat to the healthy growth of infants and young children.

Metformin is a commonly prescribed pharmacological agent for the therapy of type 2 diabetes mellitus [6]. Extensive research has revealed its potential therapeutic effects in various conditions, including polycystic ovary syndrome [7], cardiovascular disease [8], cancers [9,10], and even life extension and hair growth promotion [11]. In recent years, limited studies conducted both domestically and internationally have indicated that metformin may possess inhibitory effects against viral infections. Specifically, it has demonstrated protective effects against hepatitis C virus and hepatitis B virus infections [12]. During the virus infection, it demonstrated that metformin can inhibit complex I of the mitochondrial electron transport chain, leading to a reduction in ATP production and an increase in AMP levels, and pre-treating cells with metformin before viral infection can inhibit viral replication and protein synthesis, significantly reducing viral titers [13,14], indicating the great potential of metformin as a general anti-virus agent. It is indicated that metformin hydrochloride not only plays a role in liver function but also in intestinal function [15], and its uptake in the intestinal epithelial cells is mediated by OCTs [16].

During the peak season, over 90% of diarrhea cases in infants and young children are caused by A-type rotavirus infection. After a rotavirus infection, the absorption of glucose in the small intestine decreases, resulting in osmotic diarrhea [17]. The oral administration of metformin can impact D-glucose metabolism in the small intestine, leading to alterations in D-glucose absorption and basal lateral D-glucose uptake in intestinal tissue [18]. Inspired by this, there is an attractive hypothesis regarding whether metformin can inhibit rotavirus infection. Generally, the study of virus infections has traditionally relied on the use of cell lines and animal models. Cell models offer advantages such as easy operation, low cost, and quick detection. However, they lack the ability to faithfully reproduce intricate three-dimensional environments. Conversely, animal models pose challenges in terms of cost, control, and lengthy testing periods, rendering them less conducive for expedited drug development in emergency situations such as the COVID-19 pandemic. Therefore, the advent of organoid technology has revolutionized the field of infection biology, presenting significant advancements. Organoids, which are self-organizing 3D cultures, offer an alternative to traditional platforms, amalgamating the advantages of both 2D cell lines and in vivo animal models [19]. In our previous research, the small intestine organoids model was introduced to evaluate the antiviral effects of drugs such as IFN-α, ribavirin, and 6-thioguanine (6-TG) [20,21]. It has been confirmed that the small intestine organoid is a promising model for studying the interaction between viruses and hosts, as well as for evaluating antiviral drugs. Since the onset of the COVID-19 pandemic, diverse organoid systems have been employed as expeditious, effective, and precise tools to investigate the biology of SARS-CoV-2, assess the efficacy of prospective medications, and comprehend cellular tropism [22,23]. It is worth noting that metformin has been reported to have therapeutic potential in COVID-19. Metformin reduces blood glucose levels and may also inhibit virus infection and replication by suppressing the translation of viral proteins. Additionally, it is reported to have a regulatory effect on inflammation and immune response in COVID-19 patients [24].

During the epidemic season, the infection rate of type A rotavirus, as a typical serotype, induces over 90% diarrhea, especially in infants. Therefore, we selected simian rotavirus strain SA11, a representative A rotavirus with clear characteristics, which is a classic and widely used strain for the infection and anti-infection models. Hence, we examined the impact of metformin hydrochloride on the replication of rotavirus using in vitro two-dimensional Caco2 cell culture, small intestine organoids, and in vivo studies involving suckling mice.

## 2. Results

### 2.1. Rotavirus Causes Morphology Changes in Caco2 and Organoids

**Figure 1:**
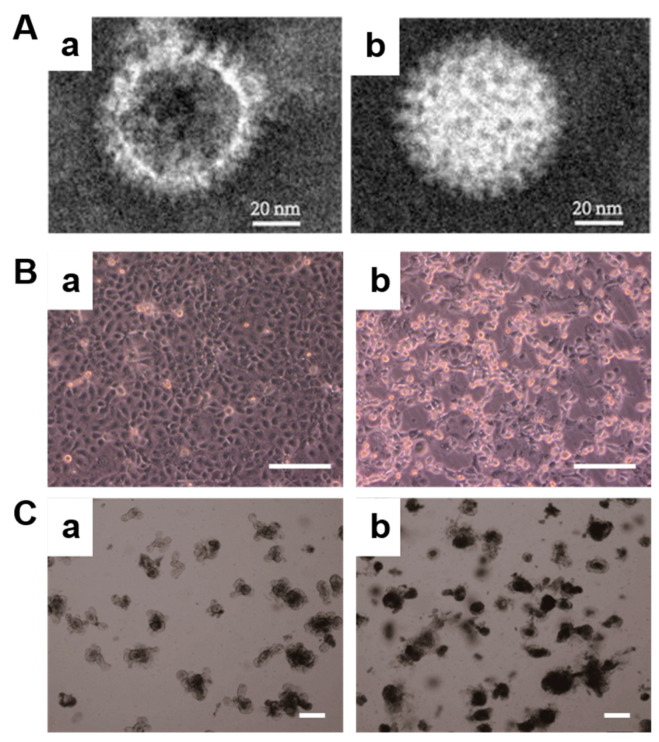
Morphology of rotavirus and infected cells and organoid. (**A**) TEM images of rotavirus SA11 hollow virus particles (a) and solid virus particles (b); (**B**) bright-field images of before (a) and after (b) the infection of Caco2 cells with rotavirus, Bar = 200 μm; and (**C**) bright-field images of before (a) and after (b) the infection of organoids with rotavirus, Bar = 200 μm.

Transmission electron microscopy (TEM) images of rotavirus SA11 exist in two common forms. One form is characterized by hollow virions (Figure 1A(a)), while the other form consists of solid virions (Figure 1A(b)), which is consistent with previous findings [25]. The RV SA11 exhibits a well-defined morphology, resembling a wheel-like structure with spoke-like features, measuring approximately 60 nm in diameter. The presence of incomplete or defective virus particles, such as the hollow virus particles observed in this study, suggests the possibility of viral destruction and the loss of nucleic acids. Upon observing cells infected with rotavirus, normal cells are densely packed and exhibit clear boundaries (Figure 1B(a)). However, after 48 h of rotavirus infection, noticeable damage can be observed (Figure 1B(b)). The intercellular gaps widen, and individual cells gradually shrink into a distinct shiny circular shape, indicating significant differences in morphology.

In this study, we established an innovative in vitro 3D model utilizing mouse small-intestine-derived organoids. The primary intestinal crypt was isolated and cultured in the presence of Matrigel and growth factors, resulting in the formation of small intestinal organoids (Figure S1). The mature organoids exhibited multiple buds, resembling the structure of intestinal villi. Following fluorescence staining of the cytoskeleton, it was observed that multiple cells were arranged in order and organically combined to form a 3D microstructure (Figure S2). After two days of subculture, the organoid displayed a clear margin, and the buds exhibited healthy growth (Figure 1C(a)). However, upon infection with rotavirus, the growth rate of the organoid decreased. An accumulation of metabolic waste was observed within the central cavity of the organoid, resulting in a relatively dark coloration (Figure 1C(b)).

### 2.2. Toxicity of Metformin Hydrochloride to Cells and Organoids

**Figure 2:**
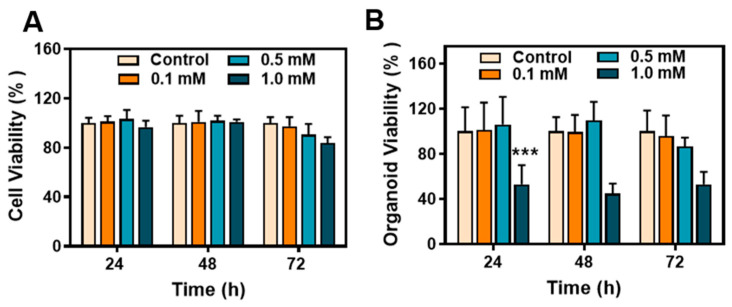
Biocompatibility and antiviral effect of metformin hydrochloride. (**A**) Cell viability of different concentrations of metformin hydrochloride in Caco2 cells after 24, 48, and 72 h; (**B**) cell viability of different concentrations of metformin hydrochloride in organoids after 24, 48, and 72 h. *** *p* < 0.001.

To assess the toxicity of metformin hydrochloride in cells and organoids, we examined three time points (24, 48, and 72 h) and various drug concentrations. In the early stages, cell viability remained unaffected across different drug concentrations. However, after 72 h of treatment with a 1.0 mM drug concentration, slight toxicity was observed in the cells (Figure 2A). Overall, the cytotoxicity of the drug was not significant. In the organoid model, exposure to a concentration of 1.0 mM of metformin hydrochloride resulted in certain toxicity, leading to a significant decline in the growth activity of the organoids. However, lower concentrations of the drug did not have a noticeable impact on organoid growth (Figure 2B).

This experiment revealed distinct toxicity patterns between cells and organoids when exposed to the drug, indicating variations in drug evaluation between 2D cell cultures and 3D organoid models. The difference between the models suggests that organoids exhibit a higher sensitivity to drugs than cells.

### 2.3. Metformin Hydrochloride Inhibited Rotavirus Gene and Protein Expression in Caco2 Cells

To assess the impact of metformin hydrochloride on rotavirus replication, we exposed SA11 rotavirus-infected Caco2 cells to varying concentrations (0, 0.1, 0.5, 1.0 mM) of the drug for 48 and 72 h. As a result, this treatment exhibited a dose-dependent cytopathic effect on Caco2 cells (Figure S3). At 24 h, the effects of metformin hydrochloride on SA11 in Caco2 cells were not apparent, and no significant differences in the cell growth state were observed under a microscope. However, changes in gene expression may have already begun. One study examined the relative abundance of over 8000 human transcripts in Caco-2 cells infected with rotavirus, and found that 6.7% of the analyzable transcripts underwent changes at 16 h post infection. Among them, 73.4% of the genes were upregulated, and these genes are involved in the dynamic regulation of protein phosphorylation, calcium homeostasis, inflammation, and cellular cytoskeleton components [26]. The obvious morphological changes in Caco2 were observed 48 h after infection. As the concentration of metformin hydrochloride increased, fewer cells exhibited a rounded morphology. By 72 h, most of the cells in the control group had developed lesions and underwent severe detachment, leading to blurred cell boundaries. However, with the increase in metformin hydrochloride concentration, the number of exfoliated cells gradually decreased.

**Figure 3:**
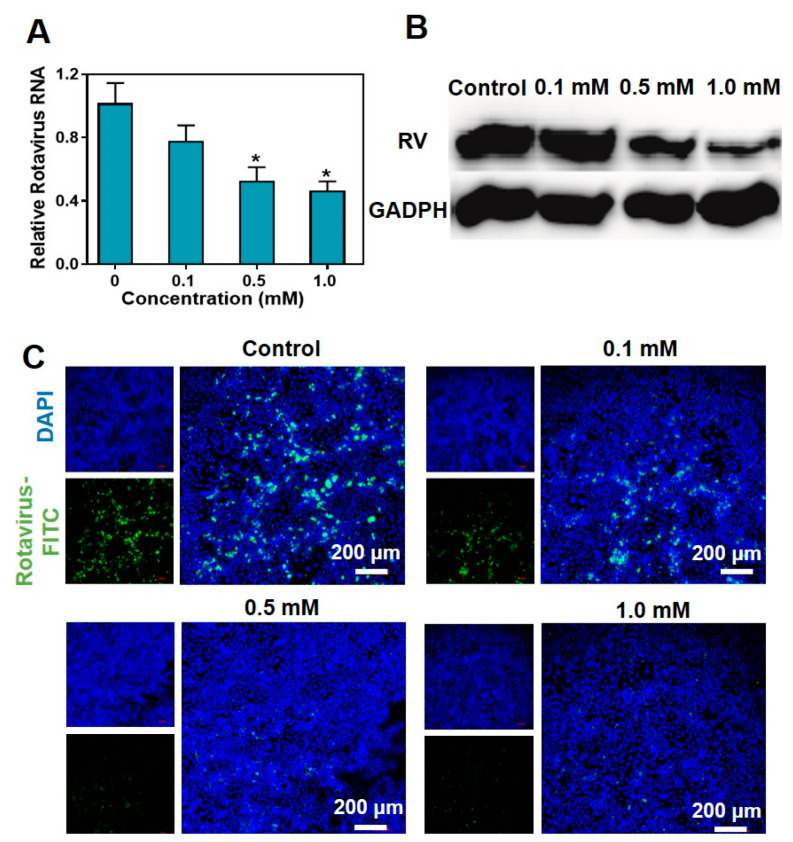
Antiviral effects of metformin hydrochloride in different concentrations in Caco2 cells. (**A**) Treatment with metformin hydrochloride (48 h) significantly inhibited viral RNA in rotavirus-infected Caco2 cells (*n* = 6, means ± SEM, * *p* < 0.05); (**B**) treatment with metformin hydrochloride (48 h) significantly suppressed viral protein expression in Caco2 cells infected with SA11 rotavirus; (**C**) laser confocal characterization of antiviral effects in Caco2 cells. Blue: DAPI, green: FITC labeled rotavirus.

To further verify the impact of metformin hydrochloride on rotavirus replication, we subjected SA11 rotavirus-infected Caco2 cells to various concentrations of metformin hydrochloride for 48 h. The relative RNA levels of viral genomic RNA were then assessed. The results demonstrated that metformin hydrochloride significantly inhibited rotavirus RNA replication in a dose-dependent manner (Figure 3A). Specifically, treatment with 0.5 and 1.0 mM concentrations of metformin hydrochloride resulted in a 0.53 ± 0.09- and 0.46 ± 0.06-fold decrease in SA11 rotavirus mRNA, respectively.

To investigate whether the reduction in SA11 gene expression correlates with a decrease in virus protein content in the cell culture system, we conducted a Western blot assay to evaluate the levels of virus proteins in SA11 rotavirus-infected Caco2 cells following a 48 h treatment. Using an anti-VP6 antibody, a specific rotavirus protein of approximately 45 kDa was detected. As shown in Figure 3B, after treatment with 0.5 mM and 1.0 mM of metformin hydrochloride, the bands corresponding to rotavirus proteins exhibited reduced width and depth. Finally, the rotavirus labeled with FITC fluorescence was visualized under confocal laser scanning microscopy (CLSM). As the concentration of metformin hydrochloride increased, the amount of viral fluorescence decreased (Figure 3C). This finding further confirms that metformin hydrochloride has a significant impact on the expression of rotavirus.

### 2.4. Metformin Hydrochloride Inhibited Rotavirus Gene and Protein Expression in Organoids

After establishing the inhibitory effect of metformin hydrochloride on rotavirus replication in 2D cell cultures, the investigation was extended to examine its impact on rotavirus replication in a 3D organoid model.

**Figure 4:**
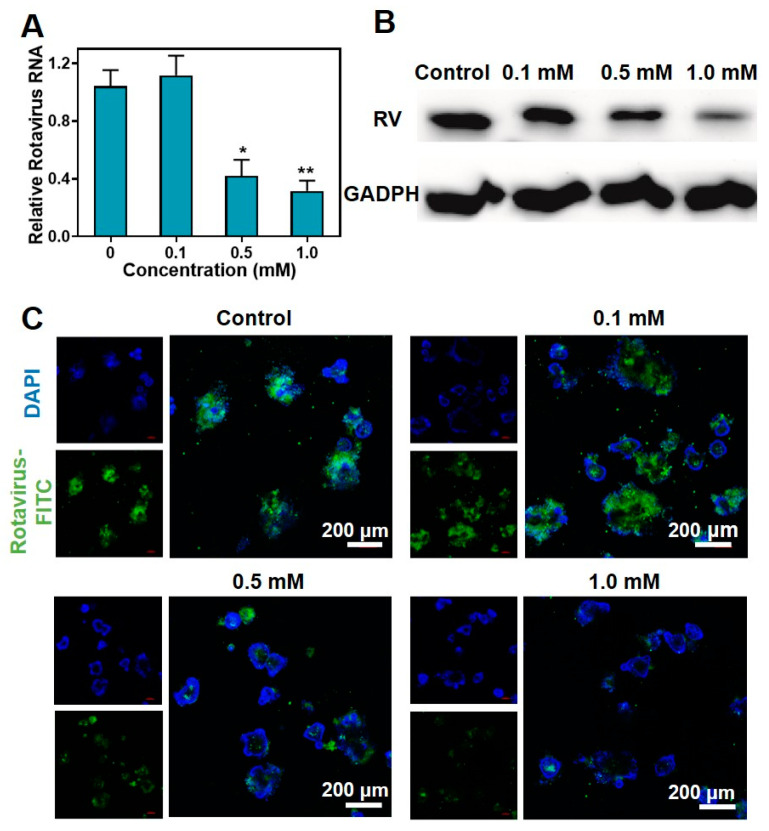
Antiviral effects of metformin hydrochloride in different concentrations in intestinal organoids. (**A**) Metformin hydrochloride treatment (48 h) significantly suppressed viral RNA levels in intestinal organoids infected with rotavirus (*n* = 6, means ± SEM, * *p* < 0.05, ** *p* < 0.01); (**B**) treatment with metformin hydrochloride (48 h) significantly inhibited viral protein in SA11 rotavirus-infected intestinal organoids; (**C**) antiviral effects in intestinal organoids. Blue: DAPI, green: FITC labeled rotavirus.

The rotavirus-infected intestinal organoids were treated with metformin hydrochloride for 48 h, and then the expression of the SA11 gene was assessed using RT-qPCR. The results revealed that treatment with 0.5 and 1.0 mM of metformin hydrochloride led to a decrease in SA11 rotavirus mRNA levels, with fold reductions of 0.418 ± 0.113 and 0.317 ± 0.017, respectively (Figure 4A). Additionally, Western blot analysis confirmed the inhibitory effect of metformin hydrochloride on rotavirus replication at concentrations of 0.5 and 1.0 mM (Figure 4B). Immunofluorescence staining demonstrated a decrease in virus fluorescence intensity as the concentration of metformin hydrochloride increased (Figure 4C). These results confirm the antiviral efficacy of metformin hydrochloride in the organoid model.

### 2.5. Metformin Hydrochloride Inhibits the Expression of the Virus in Suckling Mice

**Figure 5:**
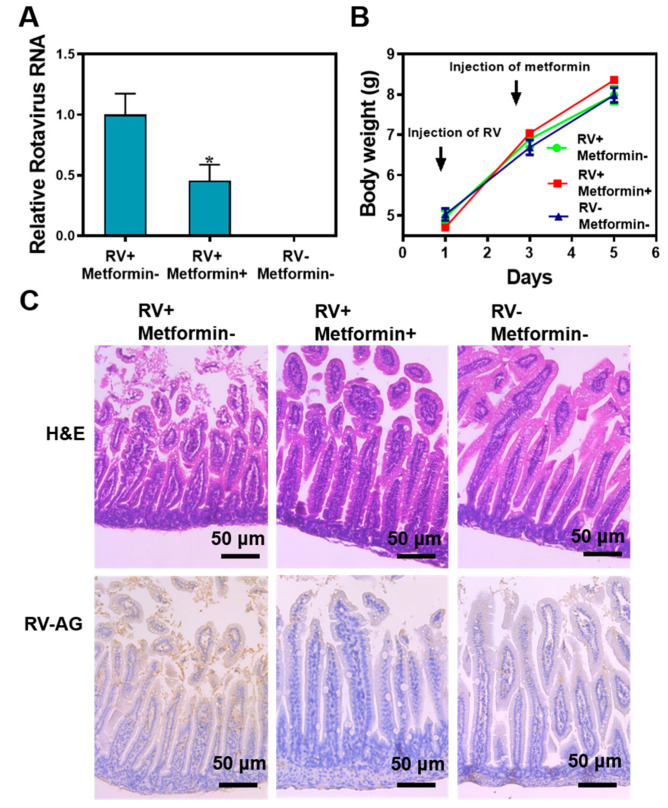
Anti-rotaviral effects of metformin hydrochloride in suckling mice. (**A**) Treatment with metformin hydrochloride (48 h) significantly inhibited viral RNA in rotavirus-infected suckling mice (*n* = 7, means ± SEM, * *p* < 0.05); (**B**) sucking mice weight measurement before and after treatment with metformin; (**C**) H&E and IHC staining images of the small intestine after virus infection and after metformin treatment.

To further verify the antiviral efficacy of metformin hydrochloride in vivo, a suckling mice model infected with rotavirus was established. The mice were weighed before and after the injection of the virus and metformin hydrochloride. Blood glucose levels were monitored following the administration of metformin hydrochloride. Notably, when rotavirus was intraperitoneally injected into the suckling mice, diarrhea was not evident, and the amount of virus transmitted to the intestinal tract via intraperitoneal injection may be relatively lower. The expression of rotavirus in the small intestine was evaluated using RT-qPCR in three different groups. The results revealed that the group treated with metformin hydrochloride exhibited less than a 0.5-fold (n = 7, *p* < 0.01) increase in viral RNA compared to the virus-infected group (Figure 5A). No expression of the rotavirus gene was detected in the mock control group, indicating that metformin hydrochloride effectively suppresses rotavirus gene expression in the small intestine of suckling mice. Figure 5B shows that there were no significant differences in body weight among the three groups during the entire experiment. However, the experimental group displayed a faster weight gain compared to the blank control group. This observation could be attributed to the increased consumption of breast milk by the suckling mice due to the injection-induced fear response after each administration.

HE staining revealed that the intestinal epithelium of mice infected with rotavirus exhibited noticeable vacuolar degeneration, accompanied by swelling and shedding of villi (Figure 5C). In contrast, the metformin hydrochloride treatment group displayed relatively intact intestinal epithelium and villi compared to the infected group. Immunohistochemistry (IHC) results demonstrated that the blank control group and the drug-treated group exhibited either negative or weakly positive virus infection signals. In the RV group, there was a significant aggregation of brown-yellow particles around the vacuolar cells of the small intestine epithelial cells, indicating positive rotavirus infection. Conversely, in the metformin hydrochloride-treated group, the replication of rotavirus in the small intestine was inhibited, resulting in a reduced degree of infection. These findings provide strong evidence that metformin hydrochloride effectively inhibits rotavirus replication in the small intestine and mitigates the extent of infection.

## 3. Discussion

In this study, we have presented convincing evidence of the suppressive effect of metformin hydrochloride on rotavirus replication, both in vivo and in vitro. The presence of rotavirus poses a significant risk to individuals who have undergone organ transplants or suffer from inflammatory bowel disease (IBD), although newly discovered medications necessitate further assessment. Metformin hydrochloride is one of the most commonly used hypoglycemic drugs in clinics, and it represents a logical choice for managing postoperative hyperglycemia in organ transplant patients who face the potential threat of rotavirus infection.

Our findings established that the current study highlights the antiviral properties of metformin hydrochloride. For instance, prior research has indicated that metformin treatment effectively suppresses the expression of hepatitis B surface antigen (HBsAg) and demonstrates the moderate inhibition of HBV replication and HBeAg expression in in vitro studies [12]. Furthermore, research has demonstrated that metformin administration in HIV-infected males results in a decrease in plasma concentration in monocyte chemoattractant protein-1 (MCP-1). This reduction in MCP-1 levels is associated with elevated fasting and postprandial cholesterol levels, as well as the increased activity of paraoxonase (PON1). This, in turn, reduces the post-prandial proinflammatory response and improves fasting and postprandial antioxidant capacity [27]. As a result, metformin can ameliorate issues related to lipodystrophy, insulin resistance, and atherosclerosis caused by HIV.

In our study, we initially infected two-dimensional Caco2 cells with rotavirus and confirmed the inhibitory effect of metformin hydrochloride on rotavirus replication in these cells. To further investigate the antiviral potential of metformin, we developed a model using rotavirus-infected small intestinal organoids. We observed that dimethylhydrazine hydrochloride still exhibited an inhibition of rotavirus in the organoids, but high concentrations of metformin hydrochloride showed some toxicity, consistent with known gastrointestinal side effects associated with high doses of metformin hydrochloride [28]. Unlike Caco2 cells, organoids displayed more sensitivity to different concentrations of metformin hydrochloride, indicating that organoids overcome the inherent limitations of two-dimensional immortalized cell lines as a biological model. Small intestinal organoids are expanding, self-organizing structures resembling the crypt-villus morphology and cellular composition of the in vivo small intestinal epithelium. They are generated from isolated small intestine crypts in a medium containing R-spondin 1, EGF, and Noggin [29,30]. This three-dimensional environment enables information exchange and signal transmission between cells to more closely resemble the natural environment, providing valuable insights for drug evaluation using organoids. Our previous studies have utilized primary intestine organoids as a model to investigate rotavirus infection. These studies have showcased the effectiveness of primary intestine organoids in examining interactions between rotavirus and the host, as well as assessing the efficacy of antiviral medications [20]. In this work, we further supported the inhibitory effect of metformin hydrochloride on rotavirus in the intestinal organoid model. Moreover, we observed 100% survival in suckling mice infected with rotavirus, and metformin hydrochloride exhibited anti-rotavirus activity in mice, consistent with our in vitro findings.

Metformin is widely recognized as an established oral hypoglycemic drug worldwide. It exhibits a stronger hypoglycemic effect in animals with diabetes resulting from partial pancreatic destruction compared to animals with euglycemia. In animals without diabetes, a very high dosage of metformin may be required to observe the hypoglycemic effect, which can lead to toxic side effects. This means that the clinical dosage of metformin does not induce hypoglycemia in non-diabetic patients, which serves as a fundamental principle for its utilization in various scientific research areas. However, metformin increases glucose uptake and anaerobic metabolism, which subsequently elevates lactate levels in the intestine, inhibiting liver mitochondrial glycerol phosphate dehydrogenase to reduce the conversion of lactate to pyruvate [31]. Adverse reactions in the gastrointestinal tract are attributed to elevated local concentrations of lactic acid, particularly in women and elderly diabetic patients [32]. Studies have indicated that combining metformin with other medications or using sustained-release formulations can help mitigate adverse gastrointestinal effects by preventing local drug accumulation and improving drug distribution [33]. Whether through combination therapy or altered dosage forms, it is crucial to regulate the local dosage in the intestinal tract. Thus, the safety of metformin is dose dependent. In this study, we also verified the toxicity of metformin on organoids. In suckling mice, the metformin hydrochloride group did not exhibit hypoglycemic symptoms since the suckling mice were not a diabetic model, and metformin hydrochloride did not display hypoglycemic effects, consistent with previous reports stating that metformin does not affect blood glucose levels in non-diabetic patients.

This study provided macroscopic evidence of the anti-rotavirus effect of metformin hydrochloride. Regarding its antiviral mechanism, preliminary speculation suggests a possible association with the phosphorylation of a molecular switch protein kinase known as AMPK [34,35]. Previous studies have shown that metformin can inhibit complex I of the mitochondrial electron transport chain, leading to a reduction in ATP production and an increase in AMP levels. This activates AMPK, resulting in an elevated level of AMPK phosphorylation and a significant restriction in the expression of SARS-CoV-2 proteins in infected cells in a dose-dependent manner. Additionally, pre-treating cells with metformin before viral infection can significantly reduce viral titers [13,14]. Also, the metformin could activate the Nrf2 pathway, generally inducing the cell to defend against virus infection [36]. In addition, metformin takes part in comprehensive metabolism processes within the cell, such as alterations in lipid metabolism, which could induce its antiviral effects [37].

Our study once again confirmed the feasibility of using organoids as an in vitro model for virus infection. The recent announcement by the FDA, stating that animal testing is no longer a requirement for new drug development [38], provides significant policy support for the utilization of organoids and organ-on-chip systems in antiviral drug and vaccine research and development. While there are still certain limitations associated with organoid models, efforts are continuously being made to enhance their standards. Addressing the challenges of large-scale virus infection and drug discovery research in organoids will be an important focus of future investigations.

## 4. Materials and Methods

### 4.1. Viruses and Drug Reagents

The widely used laboratory strain Simian rotavirus SA11 was employed as described before. The Simian rotavirus SA11 strain was prepared according to the method described elsewhere [20,21]. Metformin hydrochloride (Selleckchem, Houston, TX, USA) stocks (100 mM) were dissolved in PBS. All chemical substances were prepared in 25 μL aliquots and stored at −80 °C.

### 4.2. Cell Culture

The cell lines used in the study, Caco2 (a human colon cancer cell line) and MA104 (a rhesus monkey embryonic kidney cell line), were obtained from the American Type Culture Collection (ATCC). They were cultured in Dulbecco’s modified Eagle’s medium (DMEM) supplemented with 10% FBS (Gibco, Grand Island, NY, USA) and 1% Penicillin-Streptomycin (Gibco). MA104 cells were specifically employed for rotavirus amplification.

### 4.3. Culturing Primary Mouse Intestinal Organoids

Mice were euthanized by cervical dislocation, and their small intestines were excised. The stool was washed out with pre-chilled PBS. Segments of the ileum and jejunum were collected and rinsed with pre-chilled PBS to remove any remaining debris. The small intestine was then sectioned and repeatedly washed with pre-chilled PBS until the PBS became clear. Subsequently, the tissue was immersed in a 2 mM EDTA solution and incubated at 4 °C for 30 min. Following that, the tissue was again rinsed with pre-chilled PBS to eliminate any residual EDTA. The tissue was gently pipetted up and down to resuspend it, and the resulting suspension was passed through a 70 μm cell strainer. The suspension containing the crypts was centrifuged at 300× *g* for 5 min. The supernatant was discarded, and the crypts were resuspended in 10 mL of complete medium growth factor-(CMGF-) containing DMEM/F12 supplemented with 1% GlutaMAX Supplement (Gibco, Grand Island, NY, USA), 10 mM HEPES, and 1% Penicillin-Streptomycin. The suspension of crypts was collected via centrifugation at 150× *g* for 3 min. The crypts were then mixed with 50 μL of Matrigel (354231, Corning, NY, USA) in a pre-warmed 24-well plate and incubated at 37 °C with 5% CO_2_ for 15 min. Finally, 500 μL of a specialized medium for mouse intestinal organoids (IB-MI, Innovation Biotechnology, Tianjin, China) was added to each well after the Matrigel solidified.

### 4.4. Viability Assay of Cells or Organoids

Cell viability was determined using the Cell Counting Kit-8 (CCK-8) assay. Briefly, Caco2 cells (1 × 10^4^ cells/well) or organoids were plated in a 96-well plate and treated with different concentrations of metformin hydrochloride for 48 h. After that, 15 μL of CCK-8 solution was added to each well and incubated at 37 °C for 1.5 h. The absorbance at 450 nm was measured using an enzyme-linked immunosorbent assay (ELISA) reader (BIO-RAD, Hercules, CA, USA).

Further, the Cell Titer-Glo 3D cell viability assay (Promega, Madison, WI, USA) was used to quantify the vitality of organoids. For this, the medium was dropped, then pre-cold PBS was added followed by 5–10 min of oscillating reaction with a plate oscillator. After 20 min, the chemiluminescence value was detected through a chemiluminescence enzyme plate analyzer.

### 4.5. Inoculation of SA11 Rotavirus and Drug Treatment

Caco2 cells were cultured in a 10 cm dish, washed, and suspended. They were then seeded into a 48-well plate at a density of 5 × 10^4^ cells/well. Once the cell confluence reached approximately 80%, the culture medium was discarded, and the cell monolayers were washed twice with PBS. Subsequently, 100 μL of serum-free DMEM medium supplemented with 1% trypsin and SA11 rotavirus was added, and the plate was incubated at 37 °C with 5% CO_2_ for 60 min for infection. After infection, the cells were washed four times with PBS to remove free viruses. Following virus removal, the cells were treated with different concentrations of metformin hydrochloride in serum-free DMEM medium and incubated at 37 °C with 5% CO_2_. After 48 h of inoculation, the viral titer was measured using RT-qPCR.

Organoids cultured in a 24-well plate for 4 days were resuspended in pre-chilled PBS to remove Matrigel. Subsequently, 100 μL of culture medium supplemented with 1% trypsin and SA11 rotavirus was added and incubated at 37 °C with 5% CO_2_ for 90 min to induce infection. After discarding the culture medium, the organoids were washed four times to eliminate free viruses. The organoids were then resuspended in Matrigel and added back to the culture medium, followed by incubation at 37 °C with 5% CO_2_ for 15 min to allow the Matrigel to solidify. Next, 500 μL of culture medium containing different concentrations of metformin hydrochloride was added to each well, and the organoids were incubated at 37 °C with 5% CO_2_.

### 4.6. Animals

All personnel involved in this project were professionally trained. The procedures were performed in compliance with the required ethical principles, as well as relevant regulations and laws. Specific pathogen-free (SPF) BALB/c suckling mice, weighing 4.90 ± 0.07 g and aged 7 days, were procured from Shanghai Lingchang Biotechnology (Shanghai, China). The certificate number of experimental animals of Shanghai Lingchang Biotechnology is SCXK (Shanghai) 2018-0003. These suckling mice were allowed to live with their mother for normal lactation throughout the experiment. A total of 3 litters suckling mice were prepared in this research. All animals were kept under suitable feeding conditions, with compliant food and water availability. The animals were maintained at a constant temperature of 26 °C, with a humidity of 50%, and the light changed every 12 h, providing a suitable light–dark cycle for the animals. All animal procedures were performed in accordance with the Guidelines for Care and Use of Laboratory Animals of Zhejiang Sci-Tech University and approved by the Animal Ethics Committee of Zhejiang Sci-Tech University.

### 4.7. Construction of Rotavirus Infection BALB/c Suckling Mice Model

At the beginning of the experiments, animals were randomized and divided into various experimental groups, ensuring pairs of animals with similar weights (n = 7 animals per group): PBS treatment group, only virus infection group, and drug treatment group. Three litters of suckling mice were raised with their mothers and were fed normally. The mock group mice were injected with 100 μL PBS, while the control group mice were injected with 100 μL SA11 rotavirus. The experimental group mice were initially injected with SA11 rotavirus. After 48 h, the experimental group mice were further injected with metformin hydrochloride at a dose of 90 mg/kg/d. The mice were subjected to daily observations. After 48 h, the mice were sacrificed and their small intestine was collected and, respectively, preserved in −80 °C and formalin tissue fixation solution.

### 4.8. Real-Time Quantitative Reverse Transcription PCR (RT-qPCR) Analyses

Total RNA was isolated using TRIzol Plus RNA Purification Kit (Invitrogen, Carlsbad, CA, USA) according to the manufacturer’s instructions. The extracted RNA was dissolved in diethylpyrocarbonate (DEPC)-treated water, and its concentration was measured using a Nanodrop 2000C (Beckman Coulter, CA, USA). The 500 ng of total RNA was used for cDNA synthesis using the reverse transcription system from T100 (Bio-Rad) according to the manufacturer’s instructions. The resultant cDNA was diluted and used for evaluating gene expression with corresponding primers. All RT-qPCR experiments were performed by Power SYBR Green PCR Master Mix (Invitrogen, Carlsbad, CA, USA) with RT-qPCR 7500 (Invitrogen, Carlsbad, CA, USA). The expression of target mRNAs was normalized to the glyceraldehyde 3-phosphate dehydrogenase (GAPDH) mRNA. The relative mRNA levels of target genes were calculated using the 2-ΔΔCt method, where ΔΔCt = (ΔctTarget − ΔCtGAPDH) treatment − (ΔctTarget − ΔCtGAPDH) control.

### 4.9. Western Blot

Cultured cells were lysed in a lysis buffer (Beyotime, Shanghai, China) containing 0.1 M dithiothreitol (DTT) and heated for 10 min at 95 °C. The resulting cell lysates were subjected to 10% sodium dodecyl sulfate-polyacrylamide gel electrophoresis (SDS-PAGE) for 60 min, running at 150 V. Then, the proteins were transferred onto a polyvinylidene difluoride (PVDF) membrane (Immobilon-FL) for 1.5 h with an electric current of 250 mA. Subsequently, the membrane was blocked with 10 mL of blocking buffer (Beyotime Biotechnology) for 30 min at room temperature. After blocking, the membrane was incubated overnight at 4 °C with the indicated primary antibody (1:800 dilution). Then, the membrane was washed three times with PBST and PBS, respectively, followed by incubation with an HRP-conjugated goat anti-mouse IgG secondary antibody (1:5000 dilution) for 1 h with at room temperature. Following three washes, the protein bands were detected using the Odyssey 3.0 Infrared Imaging System.

### 4.10. Immunofluorescence Microscope Assay

Caco2 cells and organoids were cultured on glass-bottom dishes. After 48 h of SA11 infection, the cells and organoids were washed with PBS and fixed with 4% paraformaldehyde for 10 min. They were then blocked with a blocking buffer from Beyotime biotechnology for 60 min. Following blocking, the samples were incubated overnight at 4 °C with an anti-rotavirus antibody (ab181695) from Abcam (Cambridge, UK). Subsequently, the samples were incubated with Alexa Fluor 488-labeled goat anti-mouse secondary antibodies (Beyotime Biotechnology) at a 1:500 dilution. The nucleus was stained with DAPI (4,6-diamidino-2-phenylindole) from Beyotime biotechnology for 5 min at room temperature. Finally, the images were captured using immunofluorescence microscopy.

### 4.11. Immunohistochemistry

Preparation of sample embedding and slicing: After dehydration and paraffin embedding, the samples were subjected to alcohol dehydration at different concentrations. After embedding, they were cooled and shaped at −20 °C. The paraffin blocks were then placed in a paraffin microtome and cut into several 4 μm thick sections, which were spread onto glass slides and baked at 60 °C for 1 h. They were then stored at room temperature.

HE staining: After dehydration with different concentrations of alcohol, the paraffin sections were stained with hematoxylin-eosin (Harris) staining solution for 3–5 min, followed by a 2 min water rinse and differentiation with 0.8–1% hydrochloric acid alcohol for a few seconds, followed by another 1 min water rinse. Subsequently, the sections were immersed in eosin solution for 2 s, and then gradually dehydrated in 95% ethanol and absolute ethanol for 2 min. Finally, the sections were mounted with neutral gum for observation of the H&E staining results under a microscope.

For immunohistochemistry staining, intestinal sections (4 μm) were placed in a 0.3% hydrogen peroxide methanol solution to block endogenous peroxidase, and incubated at room temperature for 10 min in the dark. The slides were then placed in PBS (pH = 7.4) and washed three times for 3 min each on a decolorizing shaker. Antigen retrieval was performed by boiling sections in Tris/EDTA buffer (pH = 9) for 20 min, followed by cooling the sections with pre-cold PBS three times on the shaker for 3 min each. The sections were blocked with 1% BSA for 20 min at room temperature. The sections were incubated with primary antibody (Abcam 16825) diluted in 1% BSA (1:50) at 4 °C overnight. Subsequently, the sections were incubated with a secondary antibody (Dako, Carpinteria, CA, USA), and the sections were counterstained with hematoxylin for 4 min, followed by rinsing the slides with tap water. Dehydration and sealing steps were performed according to the H&E staining protocol. Finally, the slides containing the stained sections were observed using microscopy (Nikon, Amsterdam, The Netherlands).

### 4.12. Statistical Analysis

All results were presented as mean ± SEM of each sample (n = 3). The statistical significance of differences between different groups was assessed with the Mann–Whitney test using GraphPad Prism 5 (GraphPad Software Inc. (San Diego, CA, USA). The results were considered significant with differences between groups of *p* ≤ 0.05.

## 5. Conclusions

Our study successfully demonstrated that a concentration of 0.5 mM metformin hydrochloride effectively inhibited rotavirus replication without inducing adverse reactions in the intestine. These findings suggest that metformin hydrochloride has the potential to be used as a novel candidate for the treatment of rotavirus-induced diarrhea. The study may provide a reference for the clinical treatment of organ recipients and diarrhea patients who are infected with rotavirus or at risk of rotavirus replication. However, further studies are needed to elucidate the precise mechanisms underlying the inhibition of rotavirus replication.

## Data Availability

Data is contained within the article and supplementary material.

## Supplementary Materials

The following supporting information can be downloaded at: https://www.mdpi.com/article/10.3390/ph16091279/s1. Figure S1. Bright field images of intestinal organoid cultivation process: day1 (a), day3 (b), day5 (c) and day7 (d). Figure S2. CLSM images of microfilament and nuclear staining in organoid after 7 days of cultivation. Blue: DAPI, Red: microfilament. Figure S3. Bright field images of Caco2 cells infected with SA11 rotavirus and treated with different concentrations of metformin hydrochloride for 24 h and 72 h.
