# Supplementary material for: Metformin Hydrochloride Significantly Inhibits Rotavirus Infection in Caco2 Cell Line, Intestinal Organoids, and Mice
Source: Pharmaceuticals (Basel). 2023 Sep 11;16(9):1279. doi: 10.3390/ph16091279 (PMC10536476; doi:10.3390/ph16091279)
Supplement: Supplementary file 1 [file pharmaceuticals-16-01279-s001.zip › pharmaceuticals-2542734-supplementary.pdf]

Supporting Information

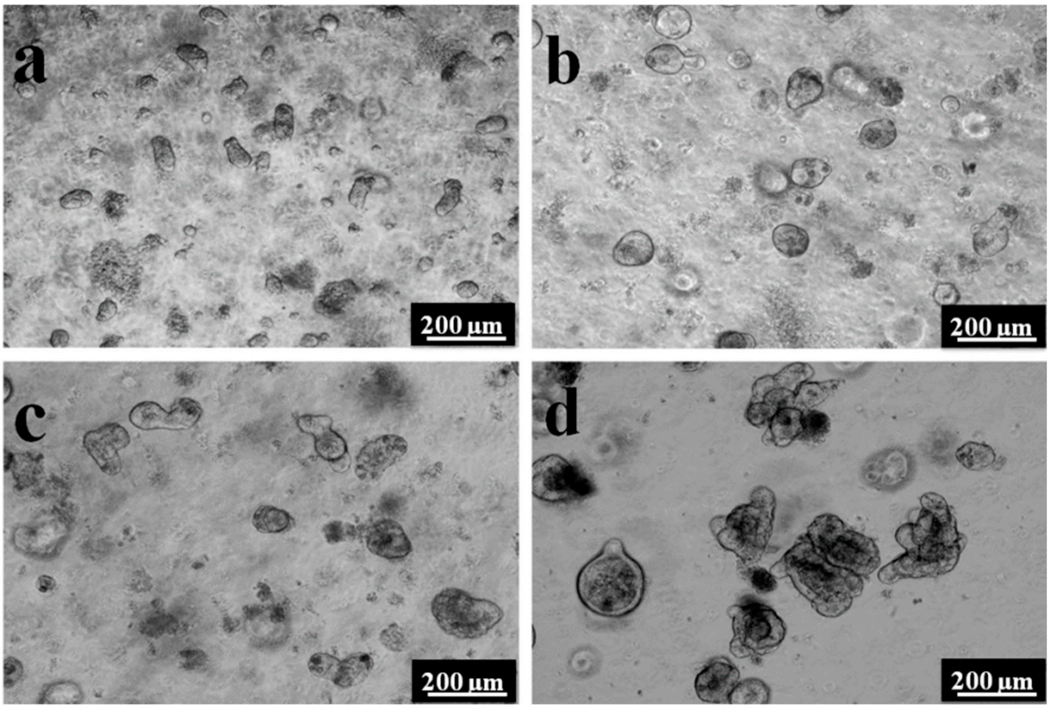

Figure S1. Bright field images of intestinal organoid cultivation process: day1(a), day3(b), day5(c) and day7(d).

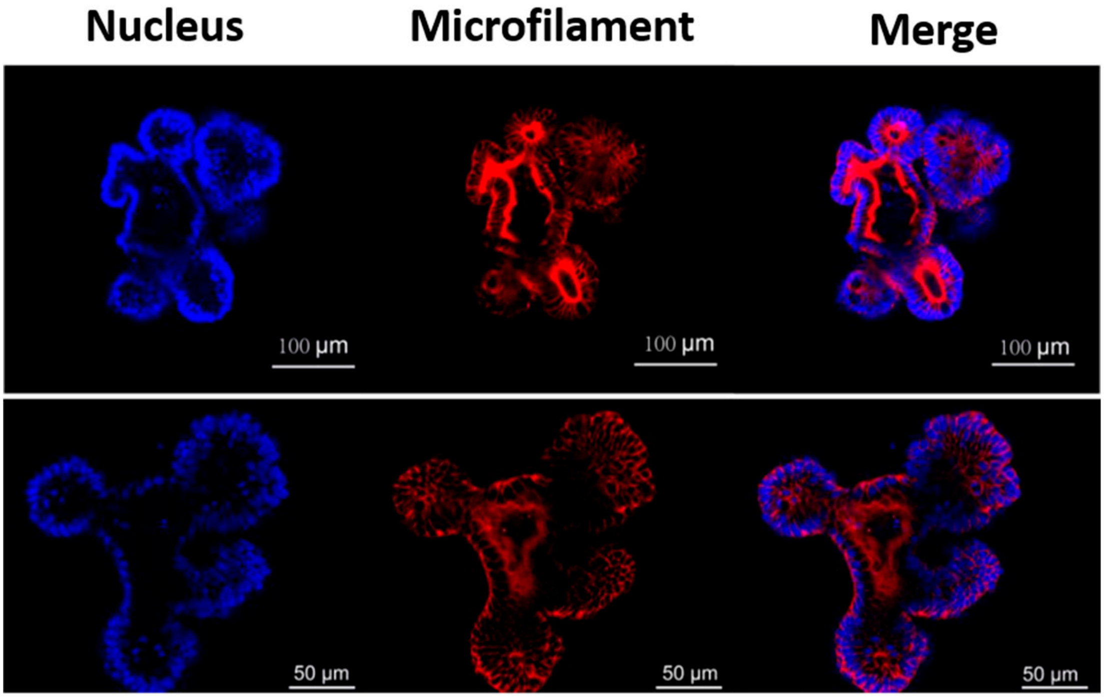

Figure S2. CLSM images of microfilament and nuclear staining in organoid after 7 days of cultivation.Blue: DAPI, Red: microfilament.

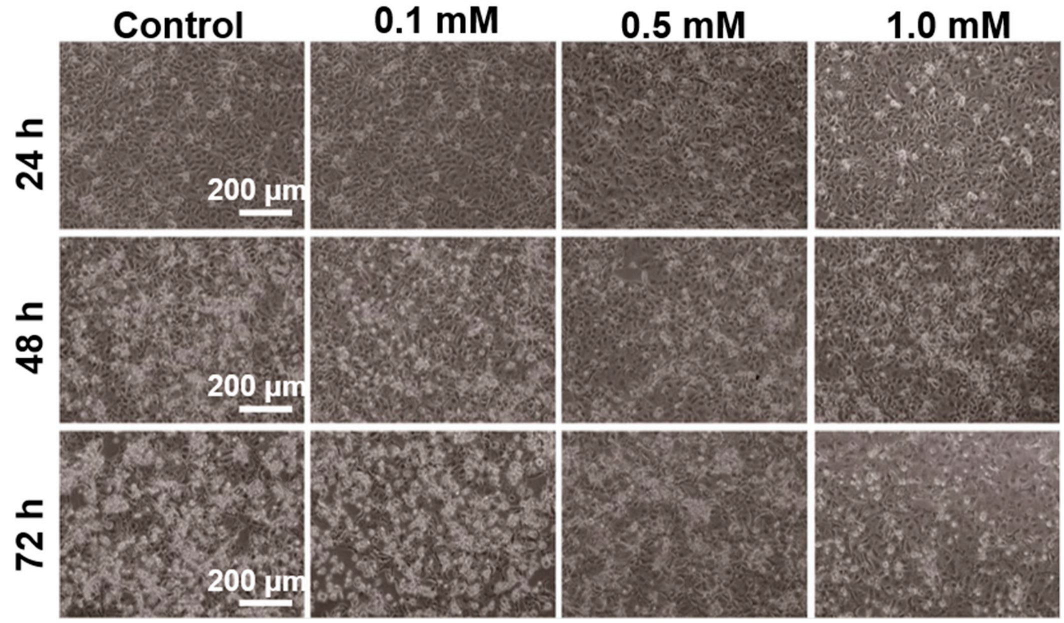

Figure S3. Bright field images of Caco2 cells infected with SA11 rotavirus and treated with different concentrations of metformin hydrochloride for 24h and 72h.
